# Early rhythm control in patients with atrial fibrillation and cancer: a subgroup analysis of the EAST-AFNET 4 trial

**DOI:** 10.1093/europace/euag269

**Published:** 2026-09-18

**Authors:** Djemail Ismaili, Anna Suling, Katrin Borof, Andreas Goette, Günter Breithardt, A John Camm, Harry Crijns, Lars Eckardt, Arif Elvan, Larissa Fabritz, Isabelle van Gelder, Michele Gulizia, Laurent Haegeli, Hein Heidbuchel, Josef Kautzner, Marc Lemoine, G André Ng, Renate B Schnabel, Lukasz Szumowski, Sakis Themistoclakis, Panos Vardas, Karl Wegscheider, Stephan Willems, Antonia Zapf, Andreas Metzner, Andreas Rillig, Paulus Kirchhof

**Affiliations:** Department of Cardiology, University Heart and Vascular Center Hamburg, Martinistraße 52, 20251 Hamburg, Germany; German Center for Cardiovascular Research (DZHK), Partner Site North, Martinistraße 52, 20251 Hamburg, Germany; Institute of Medical Biometry and Epidemiology, University Medical Center Hamburg-Eppendorf, Hamburg, Germany; Department of Cardiology, University Heart and Vascular Center Hamburg, Martinistraße 52, 20251 Hamburg, Germany; Department of Cardiology and Intensive Care Medicine, St. Vincenz Hospital, Paderborn, Germany; Medical Faculty, Otto-von-Guericke University, Magdeburg, Germany; MAESTRIA Consortium at AFNET, Münster, Germany; Atrial Fibrillation NETwork (AFNET), Mendelstr. 11, Münster 48149, Germany; Atrial Fibrillation NETwork (AFNET), Mendelstr. 11, Münster 48149, Germany; Department of Cardiology and Angiology, University Hospital Münster, Münster, Germany; Cardiology Clinical Academic Group, Molecular and Clinical Sciences Research Institute, City St. George’s University of London, Cranmer Terrace, London SW17 0RE, UK; Department of Cardiology, Cardiovascular Research Institute Maastricht, Faculty of Health, Medicine, and Life Sciences, Maastricht University, Maastricht, the Netherlands; Atrial Fibrillation NETwork (AFNET), Mendelstr. 11, Münster 48149, Germany; Department of Cardiology and Angiology, University Hospital Münster, Münster, Germany; Department of Rhythmology and Electrophysiology, Klinikum Braunschweig, Braunschweig, Germany; Department of Cardiology, University Heart and Vascular Center Hamburg, Martinistraße 52, 20251 Hamburg, Germany; German Center for Cardiovascular Research (DZHK), Partner Site North, Martinistraße 52, 20251 Hamburg, Germany; Atrial Fibrillation NETwork (AFNET), Mendelstr. 11, Münster 48149, Germany; Institute of Cardiovascular Sciences, University of Birmingham, Birmingham B15 2TT, United Kingdom; University Center of Cardiovascular Sciences, UKE, Hamburg, Germany; Department of Cardiology, University of Groningen, University Medical Centre Groningen, Groningen, The Netherlands; Cardiology Division, Garibaldi-Nesima Hospital, Catania, Italy; Department of Cardiology, University Heart Centre, University Hospital Zurich, Zurich, Switzerland; Department of Cardiology, University Hospital Antwerp, Antwerp, Belgium; Cardiovascular Research, GENCOR, Antwerp University, Antwerp, Belgium; Department of Cardiology, Institute for Clinical and Experimental Medicine, Prague, Czechia; Department of Cardiology, University Heart and Vascular Center Hamburg, Martinistraße 52, 20251 Hamburg, Germany; German Center for Cardiovascular Research (DZHK), Partner Site North, Martinistraße 52, 20251 Hamburg, Germany; British Heart Foundation Leicester Centre of Research Excellence, NIHR Leicester Biomedical Research Centre, Division of Cardiovascular Sciences, University of Leicester, Leicester, UK; Department of Cardiology, University Heart and Vascular Center Hamburg, Martinistraße 52, 20251 Hamburg, Germany; German Center for Cardiovascular Research (DZHK), Partner Site North, Martinistraße 52, 20251 Hamburg, Germany; Atrial Fibrillation NETwork (AFNET), Mendelstr. 11, Münster 48149, Germany; Arrhythmia Center, National Institute of Cardiology, Warszawa, Poland; Department of Cardiology, Dell ‘Angelo Hospital, Mestre-Venice, Italy; Department of Cardiology, Heraklion University Hospital, Heraklion, Crete 71500, Greece; Biomedical Research Foundation Academy of Athens (BRFAA), Greece and Hygeia Hospitals Group, Athens 11527, Greece; Institute of Medical Biometry and Epidemiology, University Medical Center Hamburg-Eppendorf, Hamburg, Germany; Atrial Fibrillation NETwork (AFNET), Mendelstr. 11, Münster 48149, Germany; Department of Cardiology and Intensive Care Medicine, Asklepios Hospital St. Georg, Hamburg, Germany; Institute of Medical Biometry and Epidemiology, University Medical Center Hamburg-Eppendorf, Hamburg, Germany; Department of Cardiology, University Heart and Vascular Center Hamburg, Martinistraße 52, 20251 Hamburg, Germany; Department of Cardiology, University Heart and Vascular Center Hamburg, Martinistraße 52, 20251 Hamburg, Germany; Department of Cardiology, University Heart and Vascular Center Hamburg, Martinistraße 52, 20251 Hamburg, Germany; German Center for Cardiovascular Research (DZHK), Partner Site North, Martinistraße 52, 20251 Hamburg, Germany; Atrial Fibrillation NETwork (AFNET), Mendelstr. 11, Münster 48149, Germany; Institute of Cardiovascular Sciences, University of Birmingham, Birmingham B15 2TT, United Kingdom

**Keywords:** Atrial fibrillation, Cancer, Cardio-oncology, Early rhythm control, Stroke, EAST-AFNET 4

## Abstract

**Aims:**

Early rhythm-control improves cardiovascular outcomes in patients with atrial fibrillation (AF). Its effectiveness in patients with cancer is unclear. This study evaluated the effects of cancer history on early rhythm control in the EAST-AFNET 4 trial.

**Methods and results:**

This secondary analysis of EAST-AFNET 4 compared patients with and without a history of cancer, including active cancer and cancer in remission. Outcomes included the primary outcome, a composite of cardiovascular death, stroke, or hospitalization for worsening heart failure or acute coronary syndrome; the safety outcome, a composite of death, stroke, and serious adverse events related to rhythm control; and rhythm at 24 months. Cancer history was available in 2779 of 2789 patients (99.6%). Cancer history was present in 216 of 2779 (7.8%; 19 with active cancer; mean age, 73 years; 95 [44%] women; mean CHA_2_DS_2_-VASc score, 3.5). During a follow-up of 8.6 years, early rhythm control reduced the primary outcome in patients with and without a history of cancer (*P*_interaction_ = 0.23) with high rates of sinus rhythm at 24 months (cancer: 84.9%, without cancer: 81.8%; *P*_interaction_ = 0.60). In patients with a cancer history, there was a signal for more safety outcomes with early rhythm control [early rhythm control 36/116 (31%) patients with event, 22 deaths; usual care 20/100 (20%) patients with event, 18 deaths; *P*_interaction_ = 0.055].

**Conclusion:**

Early rhythm control therapy appears effective in patients with AF and a history of cancer but may be less safe than in patients without cancer, in this hypothesis-generating analysis. This could be a play of chance but warrants further research into the safety and efficacy of rhythm control therapy in patients with a cancer history.

**Trial Registration:**

ClinicalTrials.gov Identifier: NCT01288352

## Graphical Abstract

**Graphical Abstract:**
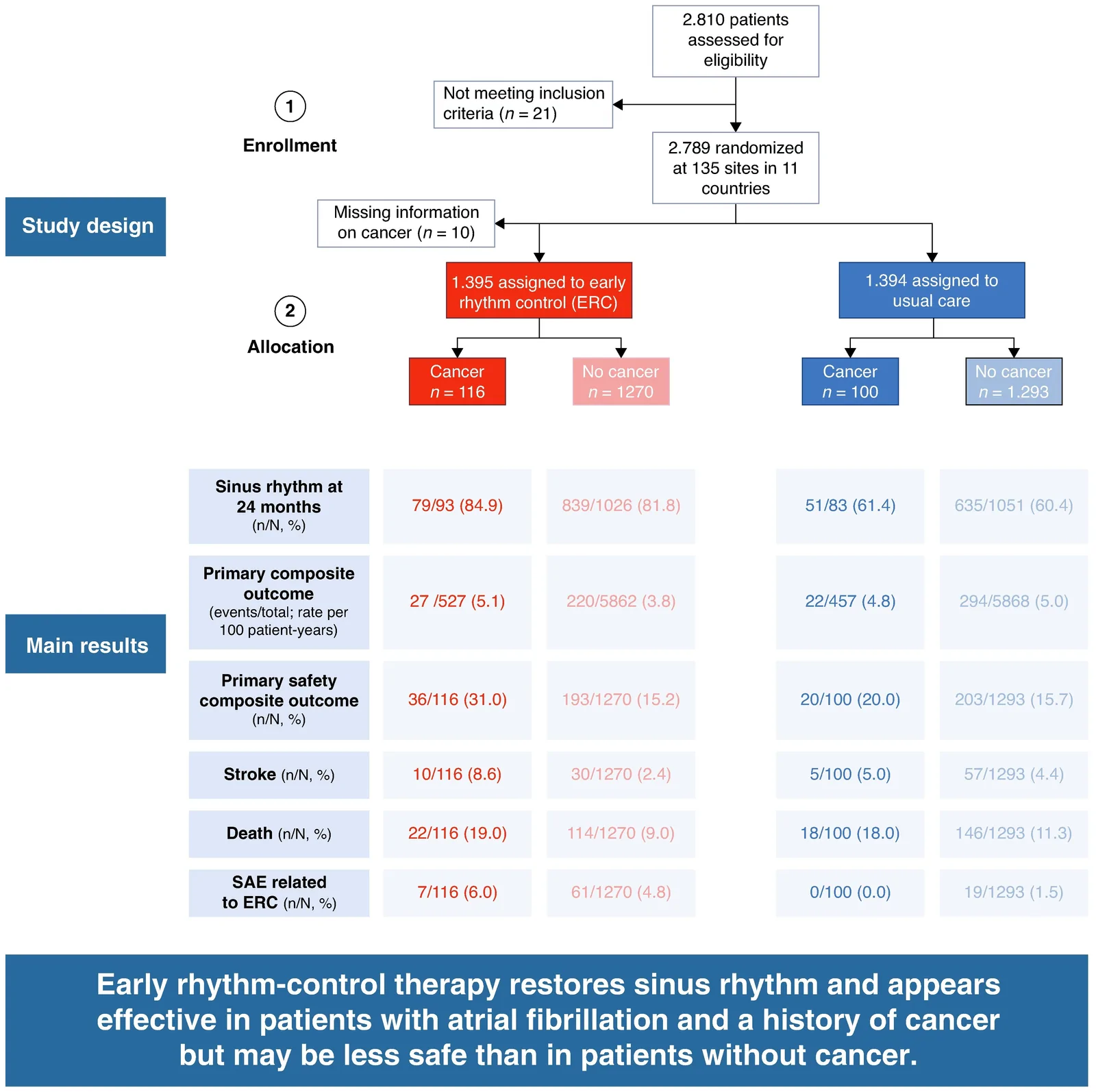

What’s new?

- Early rhythm control achieves and maintains sinus rhythm in patients with atrial fibrillation with and without a history of cancer.
- The outcome-reducing effect of early rhythm control appears to be maintained in patients with a history of cancer.
- There is a signal that early rhythm control could be less safe in patients with a history of cancer due to more strokes and more adverse events related to rhythm-control therapy.

## Introduction

Atrial fibrillation (AF), the most frequent cardiac arrhythmia, is often observed in patients with cancer.^1–3^ AF might be triggered by the malignancy itself acting on a vulnerable atrial substrate or by oncologic treatments including surgery, chemotherapy, and radiotherapy.^4–7^ Depending on the type of cancer, between one in 50 and every fourth patient with cancer has AF, compared with up to about 5%–6% in community-based populations.^5^ Patients with AF and malignant diseases appear at higher risk of thromboembolic events, heart failure, and mortality,^8–10^ while bleeding risk is also increased and complicates anticoagulation management.^11–13^ Improved cancer survival due to advances in treatment and an ageing population will increase the number of patients with AF and a history of cancer.

Rhythm control strategies in patients with cancer are complicated by drug toxicity and proarrhythmic interactions of rhythm control drugs with cancer therapy (e.g. QT-interval prolongation with antiarrhythmic drugs). Furthermore, clinicians may decide against rhythm control in view of the high comorbidity burden, frailty, and/or cancer-induced reduced quality and quantity of life and shorter life expectancy in cancer patients,^3,5,8,14^ especially when oncological treatment is considered the primary (or only) therapeutic priority.^13^ Early rhythm control therapy, mainly based on antiarrhythmic drugs, reduced a composite of cardiovascular death, stroke, or hospitalization for worsening heart failure or acute coronary syndrome in patients with recently diagnosed AF and stroke risk factors compared with usual care.^15^ The prognostic benefits of early rhythm control were reproducible across major subgroups with different cardiovascular comorbidities.^16–19^ These effects were independent of AF-related symptoms^20^ or AF pattern^21^ and were mediated by the maintenance of sinus rhythm^22^ and a reduced AF burden.^23^ The results of EAST-AFNET 4, supported by the stroke-reducing effect of dronedarone in the ATHENA trial^24^ and by observational data on the safety of early rhythm control therapy,^25,26^ initiated a paradigm shift towards rhythm control as the initial therapy in patients with recently diagnosed AF.^5^ To address the uncertainty around rhythm control therapy in cancer, this subgroup analysis of the EAST-AFNET 4 trial explored the effectiveness and safety of early rhythm control in patients with AF and a history of cancer.

## Methods

### Patients

The EAST-AFNET 4 trial was an international, multicentre, randomized controlled trial that randomized 2789 patients with recently diagnosed AF (diagnosis ≤12 months), cardiovascular comorbidities, and a life expectancy >12 months to early rhythm control therapy or usual care. The full trial protocol and primary results have been reported previously.^15,27^ All participants gave written informed consent, and the trial was approved by local ethics committees.

This subgroup analysis compared the efficacy and safety of early rhythm control in patients with and without a cancer history. Cancer history was recorded at baseline in the case report form and categorized as active malignancy (defined as malignant disease under current therapy or with currently active disease manifestation) and malignancy in remission (defined as malignant disease not under current therapy and without active disease manifestation).

Early rhythm control therapy consisted of antiarrhythmic drug therapy and/or catheter ablation, aiming to maintain sinus rhythm. If AF recurred, rhythm-control therapy was intensified by adjusting medication (e.g. adding or changing antiarrhythmic drugs) or performing ablation. In the usual care group, treatment focused primarily on rate control. Rhythm control therapies (antiarrhythmics or ablation) were used only if patients developed significant AF-related symptoms despite optimal rate control. All patients in both groups continued to receive guideline-directed therapy for stroke prevention (anticoagulation), rate control therapy, and treatment of underlying cardiovascular conditions throughout the trial.^15^

### Outcomes

The main outcomes of this analysis were the primary outcomes of EAST-AFNET 4. The first primary outcome was a composite of death from cardiovascular causes, stroke (ischaemic or haemorrhagic), or hospitalization for worsening heart failure or acute coronary syndrome. The second primary outcome was the total number of nights spent in the hospital per year, reflecting cumulative hospitalizations. The safety outcome was a composite of all-cause death, any stroke, or serious adverse events related to rhythm control therapy.^27^

### Statistical analysis

Baseline characteristics of patients with and without a history of cancer were summarized using descriptive statistics. Continuous variables are presented as mean with standard deviation (SD) or median with interquartile range (IQR) and categorical variables as frequencies and percentages. To estimate treatment effects within subgroups, an interaction term between randomized group (early rhythm control vs. usual care) and cancer history status (yes/no) was included in the model of the respective outcome. For the primary endpoint and its components, Cox proportional hazards models with a shared frailty for centre were applied, and treatment effects were expressed as hazard ratios (HRs) with 95% confidence intervals (CIs). Aalen–Johansen cumulative incidence curves were used for visualization. The second primary outcome, number of nights spent in hospital, was analysed using a mixed negative binomial model with the log of the follow-up time as offset and a random term for centre, with treatment effects presented as incidence rate ratios (IRRs). All secondary endpoints were analysed in the dataset generated by multiple imputation aligned with the main analysis of EAST-AFNET 4,^15^ assuming data were missing at random. For secondary endpoints measuring change, baseline-adjusted mixed linear models with a random term for centre were used, with treatment effects presented as mean changes. Heart rhythm at 24 months was analysed with a baseline-adjusted mixed logistic model including a random term for centre, with treatment effects expressed as odds ratios (OR). The primary safety endpoint was analysed with an analogous mixed logistic model, but without multiple imputation. All analyses were conducted according to the intention-to-treat principle, and statistical analyses were performed using validated software (StataCorp. 2025. Stata Statistical Software: Release 19. College Station, TX: StataCorp LLC.).

## Results

### Study population and baseline characteristics

Among 2779 patients randomized in the EAST-AFNET 4 trial with information on cancer (99.6% of the trial population), 216 (7.8%) had a history of cancer (active cancer in 19, prior cancer in 197 patients; *Table 1*, *Figure 1*). Compared with the 2563 patients without cancer history, patients with cancer history were older (mean age 73.4 ± 6.7 vs. 70 ± 8.4 years; *P* < 0.001) and had a slightly lower body mass index (28.3 ± 4.9 vs. 29.3 ± 5.4 kg/m^2^; *P* = 0.017, *Table 1*). The most common forms of cancer were breast cancer (24.5%), prostate cancer (21.3%), followed by skin cancer (12%), urogenital cancer (11.6%), gastrointestinal cancer (11.1%), and haematologic malignancies (10.2%). Less common were gynaecologic (4.2%), thyroid/endocrine (2.3%), head and neck (2.3%), central nervous system/neuroendocrine/thymic (1.9%), and lung/thoracic cancers (0.9%; *Table 1*). The median interval between the most recent cancer diagnosis and randomization was 6.5 years (IQR 3.0–13.5; Supplementary material online, *Table S1*).

**Figure 1:**
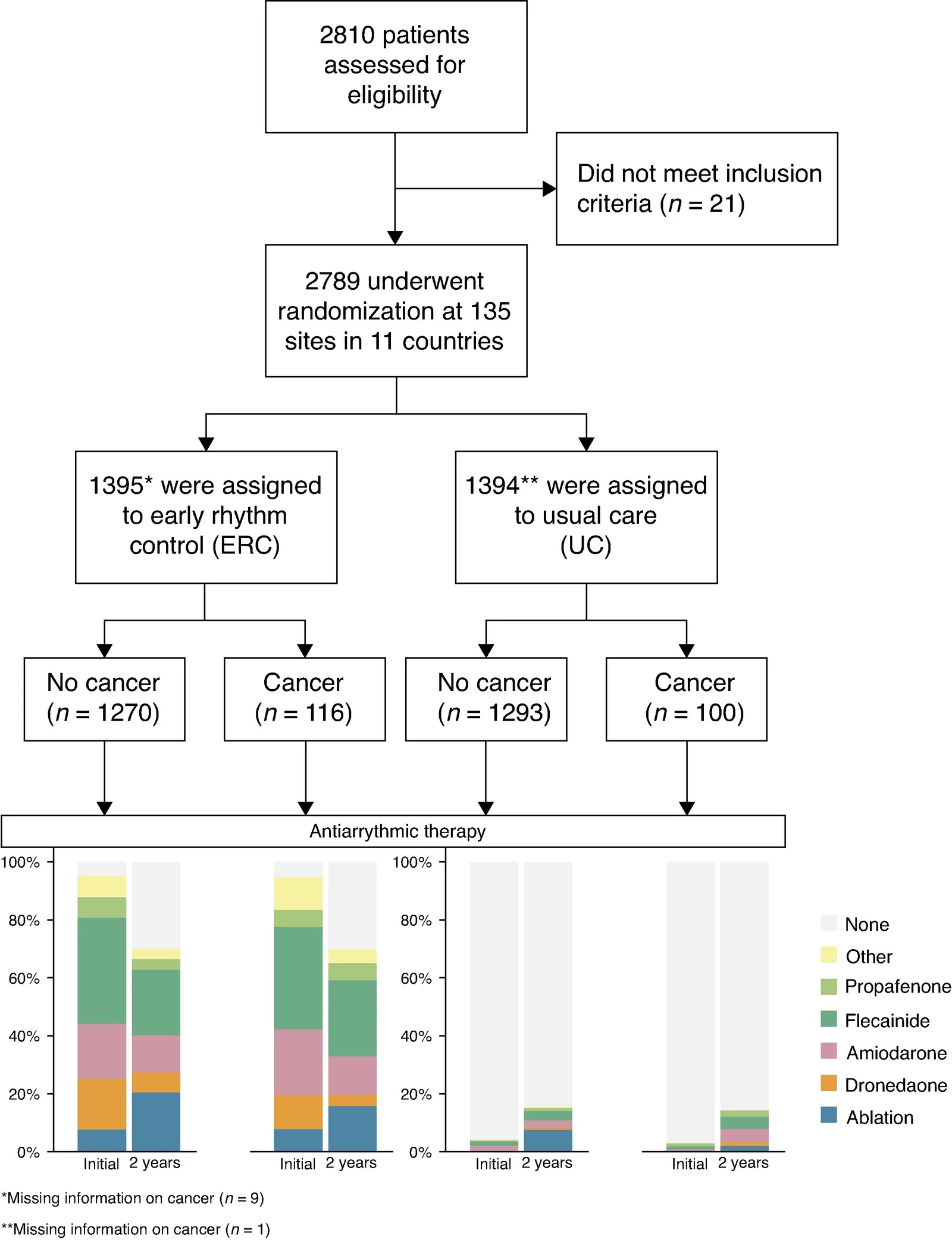
Study flow diagram. CONSORT diagram showing patient selection by cancer status and randomized treatment group.

**Table 1:** Baseline demographic and clinical characteristics of subjects by cancer history

		Cancer history		*P*-value
		No (*n* = 2563)	Yes (*n* = 216)	
Age	Mean ± SD	70.0 ± 8.4	73.4 ± 6.7	< 0.001
	Median [IQR]	71.0 [65.0; 76.0]	74.0 [69.0; 78.0]	
Gender (female)		1195 (46.6)	95 (44.0)	0.46
Body mass index (kg/m^2^) (*n* = 2768)	Mean ± SD	29.3 ± 5.4	28.3 ± 4.9	0.017
	Median [IQR]	28.7 [25.5; 32.3]	27.8 [25.0; 31.0]	
Type of AF	First episode	962 (37.5)	83 (38.4)	0.74
	Paroxysmal	920 (35.9)	72 (33.3)	
	Persistent	681 (26.6)	61 (28.2)	
Heart rhythm (sinus rhythm at baseline)		1379/2561 (53.8)	124/216 (57.4)	0.36
Days since atrial fibrillation diagnosis (*n* = 2778)	Mean ± SD	84.4 ± 184.5	74.4 ± 91.8	0.43
	Median [IQR]	36.0 [6.0; 112.0]	36.5 [5.0; 116.0]	
Absence of AF symptoms		727/2419 (30.1)	71/209 (34.0)	0.48
Previous cardioversion		997/2534 (39.3)	90/214 (42.1)	0.71
LVEF (%) (*n* = 2716)	Mean ± SD	58.8 ± 10.1	58.9 ± 8.3	0.58
	Median [IQR]	60.0 [55.0; 65.0]	60.0 [55.0; 63.0]	
Diastolic LA diameter (maximal diameter) (mm) (*n* = 2402)	Mean ± SD	43.9 ± 8.4	43.7 ± 9.1	0.50
	Median [IQR]	43.0 [38.0; 48.0]	42.0 [38.0; 48.0]	
NT-proBNP (pg/mL) (*n* = 1583)	Mean ± SD	793 ± 1212	862 ± 1093	0.50
	Median [IQR]	452 [182; 999]	458 [193; 1049]	
BMP10 (ng/mL) (*n* = 1583)	Mean ± SD	2.18 ± 0.52	2.22 ± 0.57	0.43
	Median [IQR]	2.11 [1.83; 2.43]	2.09 [1.80; 2.57]	
Concomitant conditions				
At least mild cognitive impairment (MoCA < 26)		1082/2456 (44.1)	83/206 (40.3)	0.56
Arterial hypertension		2255 (88.0%)	187 (86.6%)	0.83
Systolic blood pressure (mmHg) (*n* = 2769)	Mean ± SD	137.0 ± 19.3	136.7 ± 19.6	0.71
	Median [IQR]	135.0 [123.0; 150.0]	135.0 [120.0; 150.0]	
Diastolic blood pressure (mmHg) (*n* = 2769)	Mean ± SD	81.2 ± 12.0	79.5 ± 11.3	0.24
	Median [IQR]	80.0 [73.0; 90.0]	80.0 [70.0; 86.0]	
Stable heart failure		744 (29.0)	50 (23.1)	0.10
CHA2DS2-VASc score	Mean ± SD	3.3 ± 1.3	3.5 ± 1.3	0.35
	Median [IQR]	3.0 [2.0; 4.0]	3.0 [2.0; 4.0]	
Valvular heart disease		1136/2559 (44.4)	112/216 (51.9)	0.23
Chronic kidney disease (MDRD stage III or IV)		316 (12.3)	33 (15.3)	0.29
In patients with cancer: type of cancer (multiple answers possible)				
		Early rhythm control (*n* = 116)	Usual care (*n* = 100)	
Breast cancer		30 (25.9)	23 (23.0)	0.49
Prostate cancer		27 (23.3)	19 (19.0)	0.45
Gastrointestinal cancer		10 (8.6)	14 (14.0)	0.21
Urogenital cancer		13 (11.2)	12 (12.0)	0.89
Gynecological cancer		5 (4.3)	4 (4.0)	0.93
Thyroid/endocrine cancer		3 (2.6)	2 (2.0)	0.52
Skin cancer		13 (11.2)	13 (13.0)	0.79
Lung/thoracic cancer		0 (0.0)	2 (2.0)	—
Head and neck cancer		3 (2.6)	2 (2.0%)	0.78
Central nervous system/neuroendocrine/thymic tumors		1 (0.9)	3 (3.0)	0.28
Hematologic malignancy		14 (12.1)	8 (8.0)	0.31
In patients with cancer: cancer status				
		Active cancer	Prior cancer	
Cancer status		19/216 (8.8)	197/216 (91.2)	
Medication at discharge				
Oral anticoagulation with NOAC or VKA		2313/2561 (90.3)	199/216 (92.1)	0.65
Digoxin or digitoxin		118/2561 (4.6)	13/216 (6.0%)	0.41
Beta-blocker		2076/2561 (81.1)	169/216 (78.2)	0.30
ACE inhibitors or angiotensin II receptor blockers		1793/2561 (70.0)	135/216 (62.5)	0.012
Mineralocorticoid-receptor antagonist		167/2561 (6.5)	14/216 (6.5)	0.85
Diuretic		1035/2561 (40.4)	84/216 (38.9)	0.45
Statin		1105/2561 (43.1)	88/216 (40.7)	0.38
Platelet inhibitor		422/2561 (16.5)	32/216 (14.8)	0.37
Rhythm control at baseline	Ablation	104 (4.1)	9 (4.2)	0.71
	AAD	1162 (45.3)	104 (48.1)	
	None	1297 (50.6)	103 (47.7)	

Data are presented as mean (SD), median [IQR], or *n* (%). *P*-values were derived from mixed linear regression models for metric variables and mixed (ordinal) logistic regression models for categorical variables.

AAD, antiarrhythmic drug; ACE, angiotensin-converting enzyme; AF, atrial fibrillation; BMI, body mass index; BMP10, bone morphogenetic protein 10; CHA_2_DS_2_-VASc, Congestive heart failure, Hypertension, Age ≥75 years (doubled), Diabetes mellitus, Stroke (doubled), Vascular disease, Age 65–74 years, Sex category; IQR, interquartile range; LA, left atrium; LVEF, left ventricular ejection fraction; MDRD, Modification of Diet in Renal Disease; MoCA, Montreal Cognitive Assessment; NOAC, non-vitamin K antagonist oral anticoagulant; NT-proBNP, N-terminal pro-B-type natriuretic peptide; SD, standard deviation; VKA, vitamin K antagonist.

There was no difference in AF characteristics, including AF type, time since AF diagnosis, and AF symptoms by cancer status. Baseline left ventricular ejection fraction (LVEF), left atrial diameter, NT-proBNP, and bone morphogenetic protein 10 (BMP10) were also similar between the groups. The burden of cardiovascular comorbidities measured by the CHA_2_DS_2_-VASc score did not differ by cancer status (*Table 1*). Baseline cardiovascular medication use was also comparable, with a high use of oral anticoagulants and beta-blockers in both cohorts. The only difference was the less frequent treatment with angiotensin-converting enzyme (ACE) inhibitors or angiotensin II receptor blockers (62.5% vs. 70.0%; *P* = 0.012) in patients with a cancer history. Anticoagulation therapy and treatment of concomitant conditions remained similar at baseline and during follow-up, regardless of cancer history (*Table 2*).

**Table 2:** Oral anticoagulation at baseline and 24 months FU by cancer history and random group

	No cancer history		Cancer history	
	Early rhythm control (*n* = 1270)	Usual care (*n* = 1293)	Early rhythm control (*n* = 116)	Usual care (*n* = 100)
At discharge				
Oral anticoagulation with NOAC or VKA	1152/1269 (90.8)	1161/1292 (89.9)	111/116 (95.7)	88/100 (88.0)
All NOACs	736/1269 (58.0)	717/1292 (55.5)	62/116 (53.4)	46/100 (46.0)
Apixaban	192/1269 (15.1)	177/1292 (13.7)	8/116 (6.9)	11/100 (11.0)
Dabigatran	154/1269 (12.1)	149/1292 (11.5)	14/116 (12.1)	10/100 (10.0)
Edoxaban	23/1269 (1.8)	25/1292 (1.9)	1/116 (0.9)	0/100 (0.0)
Rivaroxaban	367/1269 (28.9)	367/1292 (28.4)	39/116 (33.6)	25/100 (25.0)
At 24 months FU				
Oral anticoagulation (NOAC and VKA) at 24 months	931/1059 (87.9)	986/1084 (91.0)	86/97 (88.7)	78/86 (90.7)
NOACs	637/1059 (60.2)	654/1084 (60.3)	52/97 (53.6)	45/86 (52.3)
Apixaban	193/1059 (18.2)	182/1084 (16.8)	14/97 (14.4)	17/86 (19.8)
Dabigatran	113/1059 (10.7)	114/1084 (10.5)	8/97 (8.2)	5/86 (5.8)
Edoxaban	26/1059 (2.5)	35/1084 (3.2)	2/97 (2.1)	1/86 (1.2)
Rivaroxaban	305/1059 (28.8)	324/1084 (29.9)	28/97 (28.9)	22/86 (25.6)

Data are presented as *n* (%).

FU, follow-up; NOAC, non-vitamin K antagonist oral anticoagulant; VKA, vitamin K antagonist.

### Rhythm control therapy

Initial rhythm control strategies planned at baseline were similar between patients with and without a history of cancer (*Table 3*, *Figure 1*). Antiarrhythmic drug therapy was the dominant rhythm control therapy, with flecainide and amiodarone being the most frequently used antiarrhythmic drugs. AF ablation as the initial therapy was planned in a similar, but small proportion of patients in both cohorts. During follow-up, ablation use increased to approximately one-fifth to one-quarter of patients assigned to early rhythm control (*Table 3*). Rhythm control therapy was initiated in about 15% of patients randomized to usual care during follow-up, without differences by cancer status.

**Table 3:** Rhythm control at baseline and 24 months FU by cancer history and random group

		No cancer history		Cancer history	
		Early rhythm control (*n* = 1270)	Usual care (*n* = 1293)	Early rhythm control (*n* = 116)	Usual care (*n* = 100)
Planned rhythm control at BL	AF ablation	102 (8.0)	2 (0.2)	9 (7.8)	0 (0.0)
	Dronedarone	218 (17.2)	1 (0.1)	14 (12.1)	0 (0.0)
	Amiodarone	247 (19.4)	27 (2.1)	26 (22.4)	1 (1.0)
	Flecainide	460 (36.2)	23 (1.8)	41 (35.3)	1 (1.0)
	Propafenone	90 (7.1)	1 (0.1)	7 (6.0)	1 (1.0)
	Other antiarrhythmic drug	93 (7.3)	2 (0.2)	13 (11.2)	0 (0.0)
	None	60 (4.7)	1237 (95.7)	6 (5.2)	97 (97.0)
Rhythm control at 24 months FU	AF ablation	243/1173 (20.7)	93/1192 (7.8)	17/106 (16.0)	2/90 (2.2)
	Dronedarone	79/1173 (6.7)	4/1192 (0.3)	4/106 (3.8%)	1/90 (1.1%)
	Amiodarone	149/1173 (12.7)	35/1192 (2.9)	14/106 (13.2)	4/90 (4.4)
	Flecainide	265/1173 (22.6)	36/1192 (3.0)	28/106 (26.4)	4/90 (4.4)
	Propafenone	47/1173 (4.0)	13/1192 (1.1)	6/106 (5.7)	2/90 (2.2)
	Other antiarrhythmic drug	40/1173 (3.4)	6/1192 (0.5)	5/106 (4.7)	0/90 (0.0)
	None	350/1173 (29.8)	1005/1192 (84.3)	32/106 (30.2)	77/90 (85.6)

Data are presented as *n* (%).

AF, atrial fibrillation; BL, baseline; ERC, early rhythm control; FU, follow-up.

### Primary and secondary efficacy outcomes

The effect of early rhythm control therapy appeared consistent in patients with and without cancer (*P*_interaction_ = 0.23). In patients without cancer, early rhythm control reduced the primary outcome rate (3.8 vs. 5.0 events per 100 person-years; HR 0.75, 95% CI 0.63–0.89; *Figure 2*). In patients with cancer, primary outcome rates were similar between treatment groups (early rhythm control 5.1 vs. usual care 4.8 events per 100 person-years). Event numbers are given in *Table 4*.

**Figure 2:**
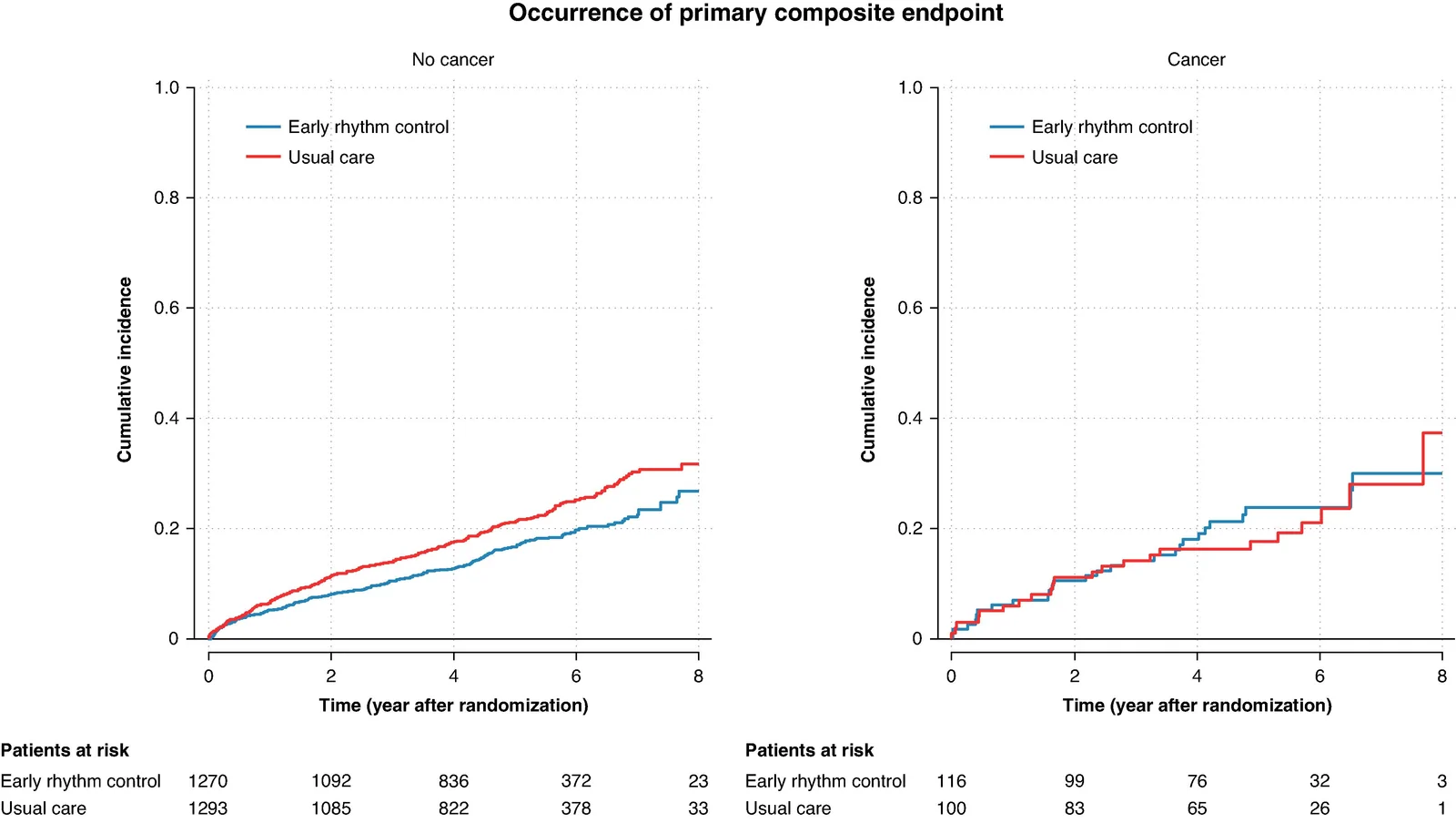
Cumulative incidence of the primary composite endpoint. Aalen–Johansen plots showing the cumulative incidence of the primary composite endpoint in patients with and without a history of cancer, comparing ERC with usual care. ERC, early rhythm control.

**Table 4:** Outcome analysis by treatment group and cancer history

	No cancer history			Cancer history			*P*-value
	Early rhythm control	Usual care	Treatment effect	Early rhythm control	Usual care	Treatment effect	
First primary outcome	220/5862 (3.8)	294/5868 (5.0)	0.75 (0.63, 0.89)	27/527 (5.1)	22/457 (4.8)	1.08 (0.61, 1.90)	0.23
Components of the first primary outcome							
Death from CV causes	58/6326 (0.9)	86/6479 (1.3)	0.69 (0.49, 0.96)	8/574 (1.4)	8/501 (1.6)	0.87 (0.32, 2.32)	0.67
Stroke	30/6240 (0.5)	57/6359 (0.9)	0.54 (0.35, 0.84)	10/557 (1.8)	5/490 (1.0)	1.72 (0.59, 5.06)	0.051
Hospitalization with wors. of HF	128/6055 (2.1)	158/6075 (2.6)	0.81 (0.64, 1.03)	10/556 (1.8)	11/476 (2.3)	0.79 (0.33, 1.87)	0.95
Hospitalization with ACS	48/6191 (0.8)	62/6316 (1.0)	0.80 (0.55, 1.16)	5/555 (0.9)	3/493 (0.6)	1.50 (0.36, 6.30)	0.40
Secondary primary outcome							
Nights spent in hospital/year	5.7 (22.5)	4.9 (15.2)	1.07 (0.94, 1.22)	6.7 (14.8)	7.2 (19.9)	1.13 (0.71, 1.79)	0.84
Key secondary outcomes at 2 years							
Change in LVEF	1.4 (9.7)	0.7 (9.8)	0.30 (−0.41, 1.01)	2.2 (9.1)	1.7 (9.4)	−0.60 (−2.95, 1.75)	0.47
Change in EQ-5D score	−0.8 (21.1)	−2.6 (22.2)	1.33 (−0.48, 3.13)	−3.2 (24.4)	−4.6 (23.7)	−0.91 (−7.19, 5.37)	0.50
Change in SF-12 Mental Score	0.7 (10.7)	1.6 (10.2)	−1.18 (−2.03, −0.32)	0.5 (9.9)	1.4 (9.0)	−1.39 (−4.23, 1.45)	0.89
Change in SF-12 Physical Score	0.3 (8.4)	0.2 (8.3)	0.33 (−0.43, 1.09)	0.3 (9.2)	−1.8 (7.0)	0.76 (−1.68, 3.20)	0.74
Change in MoCA score	0.2 (3.3)	0.1 (3.2)	−0.11 (−0.38, 0.16)	−0.5 (3.4)	0.2 (3.3)	−0.29 (−1.19, 0.62)	0.72
Sinus rhythm	839/1026 (81.8)	635/1051 (60.4)	3.08 (2.50, 3.80)	79/93 (84.9)	51/83 (61.4)	3.78 (1.79, 7.98)	0.60
Safety outcome							
Occurrence of a primary safety outcome	193/1270 (15.2)	203/1293 (15.7)	0.96 (0.77, 1.19)	36/116 (31.0)	20/100 (20.0)	1.87 (0.98, 3.56)	0.055

Numbers of events per 100 person-years are shown for the first primary outcome and its components. Treatment effects are presented as HRs, IRRs, mean differences, or ORs with 95% CIs from models including an interaction term between cancer history and randomized group. The *P*-value for interaction tests for a differential treatment effect based on cancer history status.

ACS, acute coronary syndrome; AF, atrial fibrillation; CI, confidence interval; CV, cardiovascular; EQ-5D, EuroQol 5-Dimension; HR, hazard ratio; IRR, incidence rate ratio; LVEF, left ventricular ejection fraction; MoCA, Montreal Cognitive Assessment; OR, odds ratio; SF-12, 12-Item Short Form Health Survey.

Similar to the findings in the main trial, early rhythm control did not differentially affect the second primary endpoint, nights spent in hospital per year. On average, patients without cancer spent 5.7 ± 22.5 vs. 4.9 ± 15.2 nights/year in hospital (IRR 1.07, 95% CI 0.94–1.22) and those with cancer 6.7 ± 14.8 vs. 7.2 ± 19.9 nights/year (IRR 1.13, 95% CI 0.71–1.79). Likewise, secondary outcomes at 2 years showed no relevant differences between treatment strategies in patients with a cancer history, including changes in left ventricular ejection fraction, quality of life (measured by the EuroQol 5-Dimension score), cognitive function [measured by the Montreal Cognitive Assessment (MoCA) score], and mental or physical health status (measured by the 12-Item Short Form Health Survey score).

The early rhythm control strategy proved effective in maintaining sinus rhythm with and without a history of cancer. At 24 months, 84.9% of patients with cancer history assigned to early rhythm control were in sinus rhythm, compared with 61.4% in the usual care group (OR 3.78, 95% CI 1.79–7.98). These findings were consistent in patients with and without cancer (81.8% vs. 60.4%; OR 3.08, 95% CI 2.50–3.80; *P*_interaction_ = 0.60).

### Safety outcomes

In patients without a history of cancer, the primary safety outcome occurred at similar rates in both the early rhythm control and usual care groups (193 events (15.2%) vs. 203 events (15.7%), respectively) (*Tables 4 and 5*), consistent with the overall safety profile of the main trial. This favourable safety profile was not observed in patients with a history of cancer. In patients with a history of cancer, the primary safety outcome occurred more often with early rhythm control than with usual care (early rhythm control 36 events (31.0%); usual care 20 events (20.0%); *P*_interaction_ = 0.055). All-cause mortality was similar between the treatment arms in patients with a history of cancer [early rhythm control: 22 deaths (19.0%); usual care: 18 deaths (18.0%)]. The higher event rate in patients with a history of cancer randomized to early rhythm control was attributable to more strokes [early rhythm control: 10 strokes (8.6%); usual care: 5 strokes (5.0%)] and more serious adverse events related to rhythm control therapy [early rhythm control: 7 events (6.0%); usual care: 0 events]. These events included complications such as drug-induced bradycardia, drug toxicity, and procedural events related to AF ablation (*Table 5*).

**Table 5:** Safety outcomes by cancer history and random group

	No cancer history		Cancer history	
	Early rhythm control (*n* = 1270)	Usual care (*n* = 1293)	Early rhythm control (*n* = 116)	Usual care (*n* = 100)
Occurrence of stroke	30 (2.4)	57 (4.4)	10 (8.6)	5 (5.0)
Occurrence of death	114 (9.0)	146 (11.3)	22 (19.0)	18 (18.0)
SAE of special interest related to rhythm control therapy	61 (4.8)	19 (1.5)	7 (6.0)	0 (0.0)
Serious adverse event related to antiarrhythmic drug therapy				
Non-fatal cardiac arrest	1 (0.1)	1 (0.1)	0 (0.0)	0 (0.0)
Drug-induced bradycardia	12 (0.9)	5 (0.4)	2 (1.7)	0 (0.0)
Torsade de Pointes tachycardia	1 (0.1)	0 (0.0)	0 (0.0)	0 (0.0)
Drug toxicity of AF-related drug therapy	9 (0.7)	3 (0.2)	1 (0.9)	0 (0.0)
AV nodal block	2 (0.2)	0 (0.0)	0 (0.0)	0 (0.0)
Serious adverse event related to AF ablation				
Pericardial tamponade	3 (0.2)	0 (0.0)	0 (0.0)	0 (0.0)
Blood pressure related (hypotension, hypertension, except syncope)	0 (0.0)	0 (0.0)	1 (0.9)	0 (0.0)
Syncope	3 (0.2)	1 (0.1)	1 (0.9)	0 (0.0)
Serious adverse event of special interest related to RC therapy				
Other event	1 (0.1)	3 (0.2)	0 (0.0)	0 (0.0)
Other cardiovascular event	5 (0.4)	1 (0.1)	0 (0.0)	0 (0.0)
Major bleeding	5 (0.4)	0 (0.0)	1 (0.9)	0 (0.0)
Hospitalization for AF	10 (0.8)	3 (0.2)	1 (0.9)	0 (0.0)
Bleeding caused by catheter intervention or antithrombotic therapy	1 (0.1)	2 (0.2)	0 (0.0)	0 (0.0)
Worsening of heart failure, decompensated	3 (0.2)	0 (0.0)	0 (0.0)	0 (0.0)
Implantation of a pacemaker, ICD, CRT, or any other cardiac device	8 (0.6)	4 (0.3)	0 (0.0)	0 (0.0)

Data are presented as *n* (%).

AF, atrial fibrillation; AV, atrioventricular; CRT, cardiac resynchronization therapy; ICD, implantable cardioverter-defibrillator; RC, rhythm control; SAE, serious adverse event.

## Discussion

This hypothesis-generating secondary analysis of the EAST-AFNET 4 trial provides the first randomized data on the efficacy and safety of systematic early rhythm control in patients with atrial fibrillation and a history of cancer. The analysis has two main findings:

1. Early rhythm control equally achieves sinus rhythm in patients with and without cancer in EAST-AFNET 4. The outcome-reducing effect of early rhythm control also appears to be maintained in patients with a history of cancer.
2. There is a signal that early rhythm control could be less safe in patients with a history of cancer due to more strokes and more side effects of antiarrhythmic drugs in patients with cancer. This could be a play of chance but calls for further research into the safety of rhythm control therapy in patients with a cancer history.

### Effectiveness and safety of early rhythm control in patients with and without cancer

The cancer subgroup is relatively small and heterogeneous, including various cancer types and stages, and predominantly consists of patients with cancer in remission (*Table 1*). Early rhythm control effectively restored sinus rhythm, illustrated by high rates of sinus rhythm at 2 years (>80% with and without a cancer history). Cancer and cancer therapies can aggravate atrial damage and create a persistent substrate for AF.^28–30^ The risk of AF appears to be highest early after cancer diagnosis and declines over time.^7^ Recent EAST-AFNET 4 subanalyses highlighted the prognostic importance of atrial cardiomyopathy, and a recent international clinical consensus statement emphasizes its central role in AF.^31,32^ In our cohort, however, baseline LVEF and markers of atrial cardiomyopathy, including left atrial diameter, NT-proBNP, and BMP10, were comparable between patients with and without a history of cancer. This may reflect that the cancer subgroup predominantly consisted of patients with cancer in remission rather than patients in the early phase after cancer diagnosis, when cancer- and treatment-related atrial remodelling may be most pronounced.

There was no signal that early rhythm control was less effective in patients with than without a history of cancer (*P*_interaction_ = 0.23). However, the number of strokes was not lower in patients with a cancer history, while there was a 30% reduction in stroke with early rhythm control in the overall trial (*Tables 4* and *5*).^15^ This may include chance differences and strokes not attributable to AF.^33^ Malignancy is well known to confer a prothrombotic state through mechanisms such as activation of the coagulation cascade, endothelial dysfunction, paraneoplastic processes, and lower doses or intermittent intake of anticoagulants.^34^ Patients therefore may have an increased risk of stroke independent of the presence of AF. It is conceivable that some of the strokes observed in this analysis were unrelated to AF and could potentially require stroke prevention beyond oral anticoagulation and rhythm control therapy. Oral anticoagulation use remained high at the 2-year follow-up in patients with and without a history of cancer (*Table 2*). Baseline differences between patients with and without a cancer history should also be considered. Patients with a cancer history were older than patients without cancer (*Table 1*). Older age and a high comorbidity burden alone are unlikely to explain the observed safety signal, as early rhythm control appeared to be more effective and equally safe in patients with recently diagnosed atrial fibrillation and a high comorbidity burden.^16^ Ongoing studies such as EASThigh-AFNET 11 (NCT06324188) and EAST STROKE (NCT05293080) will quantify the effectiveness and safety of rhythm control strategies in high-risk populations. Our analysis may suggest that a trial of rhythm control therapy in cancer survivors is warranted. As a substantial proportion of AF episodes in patients with cancer may remain asymptomatic,^35^ contemporary AF screening and monitoring strategies, including prolonged rhythm monitoring and wearable technologies, may facilitate earlier AF detection.^36^ Indeed, patient-operated electrocardiograms using the technologies available 15 years ago were part of early rhythm control in EAST-AFNET 4, but did not change therapy much.^23,37^ Whether contemporary rhythm monitoring translates into earlier initiation of rhythm control therapy and improved clinical outcomes in patients with cancer remains unknown and warrants prospective evaluation.

In view of the low overall event number, the numerical difference in stroke in this analysis could be due to play of chance. The findings differ from a nationwide cohort of long-term cancer survivors, where early rhythm control was associated with a 33% reduction in stroke risk.^14^ That analysis was based on a nationwide observational cohort of patients who had survived cancer for at least five years and used propensity score matching to balance treatment groups, whereas EAST-AFNET 4 was a randomized controlled trial. While the inclusion criterion of >5-year cancer survival is similar to the median duration between cancer and enrolment in EAST-AFNET 4 [median 6.5 years (IQR 3–13.5)], the present analysis also included 19 patients with active cancer (one stroke event). Furthermore, the predominance of hormonally driven malignancies (such as breast and prostate cancer) in our cohort suggests a specific biological background. Differences in tumour biology, treatment exposure, and residual prothrombotic states may partly explain the contrasting clinical outcomes observed between the two studies. Our data clearly call for further research into the interactions between different types of cancer, AF, and rhythm control therapy.

### Is antiarrhythmic drug therapy less safe in patients with a history of cancer?

There were more serious adverse events related to antiarrhythmic drugs in patients with a history of cancer than in patients without a history of cancer in this analysis. Cancer, chemotherapy, and radiation can damage the heart, and several cancer therapies are known to increase the risk of developing heart failure.^30^ Polypharmacy can further complicate rhythm management, as many anticancer drugs interact with antiarrhythmic agents or anticoagulants.^1,38,39^ These multifactorial vulnerabilities may therefore contribute to the higher rate of adverse events observed in the cancer subgroup of EAST-AFNET 4. Although our findings could be a chance finding, especially in view of the good overall safety of antiarrhythmic drugs in EAST-AFNET 4,^40,41^ the clustering of serious adverse events related to antiarrhythmic drug therapy in this analysis and the mechanisms discussed above call for caution when using antiarrhythmic drugs in patients with a cancer history and AF. These observations highlight the need for close collaboration between cardiology and oncology when considering early rhythm control strategies in patients with a cancer history and may suggest evaluating AF ablation as a safer rhythm control strategy in these patients.

## Limitations

Our study has several limitations. First, as a *post hoc* subgroup analysis with only 216 patients with a history of cancer and AF, all findings are exploratory. Furthermore, event numbers and statistical power are low. Second, the cancer category was heterogeneous, encompassing patients with active cancer and those in remission, as well as various cancer types and stages, creating heterogeneity in the patients with cancer. The cancer subgroups were too small for meaningful separate analyses. Third, although the median interval between cancer diagnosis and randomization was 6.5 years, the sample size did not allow analyses according to time since cancer diagnosis. In addition, data on specific oncologic therapies (chemotherapy, radiotherapy, targeted agents) and their timing relative to AF diagnosis were not available, precluding a more detailed assessment of the impact of cancer timing and treatment on outcomes. Furthermore, oral anticoagulation use did not differ significantly between patients with and without cancer, although it was numerically lower in cancer patients at the 2-year follow-up (88%, *Table 2*). Adherence and persistence with oral anticoagulation, important determinants of stroke prevention in patients with AF,^42^ were measured only at baseline, 1 year, and 2 years in EAST-AFNET 4. Patients with cancer may be susceptible to interruption or discontinuation of anticoagulation because of bleeding complications, thrombocytopenia, or the need for invasive procedures, which could have transiently increased thromboembolic risk and contributed to the higher rate of strokes observed in this analysis. Finally, patients with an estimated life expectancy of less than one year were excluded from the EAST-AFNET 4 trial, which likely resulted in an underrepresentation of patients with highly aggressive or advanced malignancies.

## Conclusion

Early rhythm control therapy appears effective in patients with previous or active cancer enrolled in the EAST-AFNET 4 trial. The safety of early rhythm control therapy in patients with a history of cancer warrants further evaluation, as this analysis found a possible signal for more serious adverse events related to medical and interventional rhythm control therapy. Our hypothesis-generating findings also suggest that mechanisms of stroke in patients with AF and active or previous cancer warrant further research.

## Supplementary Material

euag269_Supplementary_Data

## References

[1] Lyon AR, López-Fernánde T, Couch LS, Asteggiano R, Aznar MC, Bergler-Klei J et al 2022 ESC guidelines on cardio-oncology developed in collaboration with the European Hematology Association (EHA), the European Society for Therapeutic Radiology and Oncology (ESTRO) and the International Cardio-Oncology Society (IC-OS). Eur Heart J 2022;43:4229–361. 10.1093/eurheartj/ehac244 36017568

[2] Lehmann LH, Baldus C, Bewarder Y, Boda-Heggemann J, Bönner F, Dahm JB et al DGK-Curriculum Onkologische Kardiologie. Die Kardiologie 2025;19:389–98.

[3] Fradley MG, Beckie TM, Brown SA, Cheng RK, Dent SF, Nohria A et al Recognition, prevention, and management of arrhythmias and autonomic disorders in cardio-oncology: a scientific statement from the American Heart Association. Circulation 2021;144:E41–55. 10.1161/CIR.0000000000000986 PMC8992663 34134525

[4] Joglar JA, Chung MK, Armbruster AL, Benjamin EJ, Chyou JY, Cronin EM et al 2023 ACC/AHA/ACCP/HRS guideline for the diagnosis and management of atrial fibrillation: a report of the American College of Cardiology/American Heart Association joint committee on clinical practice guidelines. Circulation 2024;149:E1–E156. 10.1161/CIR.0000000000001193 PMC11095842 38033089

[5] Van Gelder IC, Rienstra M, Bunting KV, Casado-Arroyo R, Caso V, Crijns HJGM et al 2024 ESC guidelines for the management of atrial fibrillation developed in collaboration with the European Association for Cardio-Thoracic Surgery (EACTS). Eur Heart J 2024;45:3314–414. 10.1093/eurheartj/ehae176 39210723

[6] Vinter N, Christesen AMS, Fenger-Grøn M, Tjønneland A, Frost L. Atrial fibrillation and risk of cancer: a Danish population-based cohort study. J Am Heart Assoc 2018;7:e009543. 10.1161/JAHA.118.009543 PMC6201425 30371150

[7] Yun JP, Choi EK, Han KD, Park SH, Jung JH, Park SH et al Risk of atrial fibrillation according to cancer type: a nationwide population-based study. JACC CardioOncol 2021;3:221–32. 10.1016/j.jaccao.2021.03.006 PMC8352078 34396327

[8] Farmakis D, Parissis J, Filippatos G. Insights into onco-cardiology: atrial fibrillation in cancer. J Am Coll Cardiol 2014;63:945–53. 10.1016/j.jacc.2013.11.026 24361314

[9] Leader A, Mendelson Cohen N, Afek S, Jaschek R, Frajman A, Itzhaki Ben Zadok O et al Arterial thromboembolism in patients with AF and CHA2DS2-VASc score 0-2 with and without cancer. JACC CardioOncol 2023;5:174–85. 10.1016/j.jaccao.2022.08.014 PMC10152191 37144112

[10] El-Rayes M, Adam M, Fang J, Wang X, Jeong I, Austin PC et al The association of malignancy with stroke and bleeding in atrial fibrillation: a population-based cohort study. JACC CardioOncol 2025;7:157–67. 10.1016/j.jaccao.2024.10.014 PMC11866432 39967202

[11] Deitelzweig S, Keshishian AV, Zhang Y, Kang A, Dhamane AD, Luo X et al Effectiveness and safety of oral anticoagulants among nonvalvular atrial fibrillation patients with active cancer. JACC CardioOncol 2021;3:411–24. 10.1016/j.jaccao.2021.06.004 PMC8463723 34604802

[12] Raposeiras Roubín S, Abu Assi E, Muñoz Pousa I, Domínguez Erquicia P, Melendo Viu M, Gonzalez Bermudez I et al Incidence and predictors of bleeding in patients with cancer and atrial fibrillation. Am J Cardiol 2022;167:139–46. 10.1016/j.amjcard.2021.11.053 35027138

[13] Gulizia MM, Turazza FM, Ameri P, Alings M, Collins R, De Luca L et al Characteristics and management of patients with cancer and atrial fibrillation: the BLITZ-AF cancer registry. JACC Adv 2024;3:100991. 10.1016/j.jacadv.2024.100991 PMC11312304 39130025

[14] Han S, Lee SR, Choi EK, Kim B, Han KD, Choi J et al Early rhythm control of atrial fibrillation in cancer survivors: a nationwide population-based study. J Am Heart Assoc 2025;14:e040908. 10.1161/JAHA.125.040908 PMC12449946 40551338

[15] Kirchhof P, Camm AJ, Goette A, Brandes A, Eckardt L, Elvan A et al Early rhythm-control therapy in patients with atrial fibrillation. N Engl J Med 2020;383:1305–16. 10.1056/NEJMoa2019422 32865375

[16] Rillig A, Borof K, Breithardt G, Camm AJ, Crijns HJGM, Goette A et al Early rhythm control in patients with atrial fibrillation and high comorbidity burden. Circulation 2022;146:836–47. 10.1161/CIRCULATIONAHA.122.060274 35968706

[17] Rillig A, Magnussen C, Ozga AK, Suling A, Brandes A, Breithardt G et al Early rhythm control therapy in patients with atrial fibrillation and heart failure. Circulation 2021;144:845–58. 10.1161/CIRCULATIONAHA.121.056323 PMC8456351 34328366

[18] Jensen M, Suling A, Metzner A, Schnabel RB, Borof K, Goette A et al Early rhythm-control therapy for atrial fibrillation in patients with a history of stroke: a subgroup analysis of the EAST-AFNET 4 trial. Lancet Neurol 2023;22:45–54. 10.1016/S1474-4422(22)00436-7 36517170

[19] Metzner A, Willems S, Borof K, Breithardt G, Camm AJ, Crijns HJGM et al Diabetes and obesity and treatment effect of early rhythm control vs usual care in patients with atrial fibrillation: a secondary analysis of the EAST-AFNET 4 randomized clinical trial. JAMA Cardiol 2025;10:932–41. 10.1001/jamacardio.2025.2374 PMC12311819 40737012

[20] Willems S, Borof K, Brandes A, Breithardt G, Camm AJ, Crijns HJGM et al Systematic, early rhythm control strategy for atrial fibrillation in patients with or without symptoms: the EAST-AFNET 4 trial. Eur Heart J 2022;43:1219–30. 10.1093/eurheartj/ehab593 PMC8934687 34447995

[21] Goette A, Borof K, Breithardt G, Camm AJ, Crijns HJGM, Kuck KH et al Presenting pattern of atrial fibrillation and outcomes of early rhythm control therapy. J Am Coll Cardiol 2022;80:283–95. 10.1016/j.jacc.2022.04.058 35863844

[22] Eckardt L, Sehner S, Suling A, Borof K, Breithardt G, Crijns H et al Attaining sinus rhythm mediates improved outcome with early rhythm control therapy of atrial fibrillation: the EAST-AFNET 4 trial. Eur Heart J 2022;43:4127–44. 10.1093/eurheartj/ehac471 PMC9584752 36036648

[23] Zeemering S, Borof K, Schotten U, Obergassel J, Camm AJ, Crijns HJGM et al Estimated atrial fibrillation burden on early rhythm-control and cardiovascular events in the EAST-AFNET 4 trial. EClinicalMedicine 2025;88:103457. 10.1016/j.eclinm.2025.103457 PMC12572810 41181841

[24] Connolly SJ, Crijns HJ, Torp-Pedersen C, van Eickels M, Gaudin C, Page RL et al Analysis of stroke in ATHENA: a placebo-controlled, double-blind, parallel-arm trial to assess the efficacy of dronedarone 400 mg BID for the prevention of cardiovascular hospitalization or death from any cause in patients with atrial fibrillation/atrial flutter. Circulation 2009;120:1174–80. 10.1161/CIRCULATIONAHA.109.875252 19752319

[25] Kim D, Yang PS, You SC, Jang E, Yu HT, Kim TH et al Age and outcomes of early rhythm control in patients with atrial fibrillation: nationwide cohort study. JACC Clin Electrophysiol 2022;8:619–32. 10.1016/j.jacep.2022.02.014 35589174

[26] Dickow J, Kany S, Cardoso R, Ellinor V, Gkoutos PT, Houten GV et al Outcomes of early rhythm control therapy in patients with atrial fibrillation and a high comorbidity burden in large real-world cohorts. Circ Arrhythm Electrophysiol 2023;16:E011585. 10.1161/CIRCEP.122.011585 PMC10205477 36942567

[27] Kirchhof P, Breithardt G, Camm AJ, Crijns HJ, Kuck KH, Vardas P et al Improving outcomes in patients with atrial fibrillation: rationale and design of the early treatment of atrial fibrillation for stroke prevention trial. Am Heart J 2013;166:442–8. 10.1016/j.ahj.2013.05.015 24016492

[28] Desai MY, Jellis CL, Kotecha R, Johnston DR, Griffin BP. Radiation-associated cardiac disease: a practical approach to diagnosis and management. JACC Cardiovasc Imaging 2018;11:1132–49. 10.1016/j.jcmg.2018.04.028 30092970

[29] Mitchell JD, Cehic DA, Morgia M, Bergom C, Toohey J, Guerrero PA et al Cardiovascular manifestations from therapeutic radiation: a multidisciplinary expert consensus statement from the International Cardio-Oncology Society. JACC CardioOncol 2021;3:360–80. 10.1016/j.jaccao.2021.06.003 PMC8463721 34604797

[30] Rakisheva A, Farmakis D, Attanasio A, Genis AB, Cohen-Solal A, Gulati G et al Prevention of cancer therapy-related cardiac dysfunction and heart failure in cancer patients and survivors. A clinical consensus statement of the Heart Failure Association, the European Association of Preventive Cardiology of the ESC, and the ESC Council of Cardio-Oncology. Eur J Heart Fail 2025;27:2084–99. 10.1002/ejhf.3753 40679941

[31] Goette A, Corradi D, Dobrev D, Aguinaga L, Cabrera JA, Chugh SS et al Atrial cardiomyopathy revisited - evolution of a concept: a clinical consensus statement of the European Heart Rhythm Association (EHRA) of the ESC, the Heart Rhythm Society (HRS), the Asian Pacific Heart Rhythm Society (APHRS), and the Latin American Heart Rhythm Society (LAHRS). Europace 2024;26:euae204. 10.1093/europace/euae204 PMC11431804 39077825

[32] Goette A, Lemoine MD, Borof K, Schotten U, Breithardt G, Camm AJ et al Prevalence and severity of atrial cardiomyopathy in patients with recently diagnosed atrial fibrillation and stroke risk factors and its association with early rhythm control: a secondary analysis of EAST-AFNET 4. Europace 2025;27:euaf256. 10.1093/europace/euaf256 PMC12559886 41061672

[33] Heo JH, Yun J, Kim KH, Jung JW, Yoo J, Kim YD et al Cancer-associated stroke: thrombosis mechanism, diagnosis, outcome, and therapeutic strategies. J Stroke 2024;26:164–78. 10.5853/jos.2023.03279 PMC11164583 38836266

[34] Shoureshi P, Tan AY. Caught between a rock and a hard place: anticoagulation in atrial fibrillation patients with cancer. JACC Adv 2023;2:100611. 10.1016/j.jacadv.2023.100611 PMC11198312 38938346

[35] Boriani G, Imberti JF, Valenti A, Vitolo M, Lopez-Fernandez T, Mei DA. Asymptomatic atrial fibrillation in patients treated with BTK inhibitors in hematologic malignancies: a systematic review and metanalysis. Eur J Intern Med 2026;0:106994. 10.1016/j.ejim.2026.106994 42297696

[36] Boriani G, Imberti JF, Asteggiano R, Ameri P, Mei DA, Farkowski M et al Mobile/wearable digital devices for care of active cancer patients: a survey from the ESC Council of Cardio-Oncology. Eur Heart J Digit Health 2025;6:162–9. 10.1093/ehjdh/ztae082 PMC11914721 40110216

[37] Metzner A, Suling A, Brandes A, Breithardt G, Camm AJ, Crijns HJGM et al Anticoagulation, therapy of concomitant conditions, and early rhythm control therapy: a detailed analysis of treatment patterns in the EAST-AFNET 4 trial. Europace 2022;24:552–64. 10.1093/europace/euab200 PMC8982435 34473249

[38] Boriani G, Corradini P, Cuneo A, Falanga A, Foà R, Gaidano G et al Practical management of ibrutinib in the real life: focus on atrial fibrillation and bleeding. Hematol Oncol 2018;36:624–32. 10.1002/hon.2503 29512173

[39] Tang CPS, Lip GYH, McCormack T, Lyon AR, Hillmen P, Iyengar S et al Management of cardiovascular complications of bruton tyrosine kinase inhibitors. Br J Haematol 2022;196:70–8. 10.1111/bjh.17788 34498258

[40] Rillig A, Eckardt L, Borof K, John Camm A, Crijns HJGM, Goette A et al Safety and efficacy of long-term sodium channel blocker therapy for early rhythm control: the EAST-AFNET 4 trial. Europace 2024;26:euae121. 10.1093/europace/euae121 PMC11154137 38702961

[41] Rottner L, Lemoine MD, Eckardt L, Borof K, Camm AJ, Goette A et al Safety and efficacy of amiodarone and dronedarone for early rhythm control in EAST-AFNET 4. Clin Res Cardiol doi: 10.1007/s00392-025-02637-0 40387892

[42] Potpara T, Markovic BG, Grygier M, Genovesi S, Tzikas A, Boveda S et al Adherence to oral thromboprophylaxis in atrial fibrillation: an overview for clinicians. Europace 2025;27:euaf250. 10.1093/europace/euaf250 PMC12579324 41082504

