# Supplementary material for: Early rhythm control in patients with atrial fibrillation and cancer: a subgroup analysis of the EAST-AFNET 4 trial
Source: Europace. 2026 Sep 18;28(10):euag269. doi: 10.1093/europace/euag269 (PMC13720307; doi:10.1093/europace/euag269)
Supplement: Supplementary file 1 [file euag269_supplementary_data.docx]

**Table S1.** Years since last diagnosis at randomization

|  | | **Random group** | | **Total (N=216)** |
| --- | --- | --- | --- | --- |
|  |  | **Early rhythm control (N=116)** | **Usual care (N=100)** |  |
| Years since last diagnosis at randomization | Mean ± SD | 10.0 ± 10.4 | 10.4 ± 9.3 | 10.2 ± 9.9 |
|  | Median [IQR] | 6.0 [3.0;12.0] | 8.0 [4.0;14.5] | 6.5 [3.0;13.5] |
|  | Range | 1.0-48.0 | 1.0-42.0 | 1.0-48.0 |
|  | Missing | 64/116 (55.2%) | 52/100 (52.0%) | 116/216 (53.7%) |

**Table S2.** Nights spent in hospital per year by cancer

|  | | **Cancer** | | **Total (N=2779)** |
| --- | --- | --- | --- | --- |
|  |  | **No (N=2563)** | **Yes (N=216)** |  |
| Nights spent in hospital per year | Mean ± SD | 5.3 ± 19.2 | 6.9 ± 17.3 | 5.4 ± 19.0 |
|  | Median [IQR] | 1.3 [0.1;4.2] | 1.7 [0.5;5.0] | 1.3 [0.1;4.3] |
|  | Range | 0.0-365.0 | 0.0-129.8 | 0.0-365.0 |

**Access to Study Protocol and Statistical Analysis Plan**

Study protocol: https://www.nejm.org/doi/suppl/10.1056/NEJMoa2019422/suppl_file/nejmoa2019422_protocol.pdf

Analysis plan: https://www.nejm.org/doi/suppl/10.1056/NEJMoa2019422/suppl_file/nejmoa2019422_appendix.pdf
